# Influenza A virus strain PR/8/34, but neither HAM/2009 nor WSN/33, is transiently inhibited by the PB2-targeting drug paliperidone

**DOI:** 10.1007/s00705-022-05696-0

**Published:** 2023-01-13

**Authors:** Georgios-Dimitrios Panagiotidis, Christin Müller, Marco Binder, Friedemann Weber

**Affiliations:** 1 Institute for Virology, FB10-Veterinary Medicine, Justus-Liebig University, 35392 Giessen, Germany; 2 Institute for Medical Virology, FB11-Medicine, Justus-Liebig University, 35392 Giessen, Germany; 3 German Center for Infection Research (DZIF), Partner Site Giessen, Germany; 4 Research Group “Dynamics of early viral infection and the innate antiviral response”, Division “Virus-Associated Carcinogenesis”, German Cancer Research Center (DKFZ), Heidelberg, Germany

## Abstract

Influenza A virus (FLUAV) is a significant human pathogen. In silico structural analysis (PMID 28628827) has suggested that the FDA-approved drug paliperidone interferes with the binding of the FLUAV polymerase subunit PB2 to the nucleoprotein NP. We found that paliperidone inhibits FLUAV A/PR/8/34 early after infection of canine MDCK II, human A549, and human primary bronchial cells, but not at late time points. No effect was detectable against the strains A/Hamburg/05/2009 and A/WSN/33. Moreover, paliperidone indeed disturbed the interaction between the PB2 and the NP of A/PR/8/34 and reduced early viral RNA and protein synthesis by approximately 50%. Thus, paliperidone has measurable but transient and virus-strain-restricted effects on FLUAV.

**Supplementary Information:**

The online version contains supplementary material available at 10.1007/s00705-022-05696-0.

Infections with influenza A virus (FLUAV; family *Orthomyxoviridae*) annually cause 3–5 million cases of severe disease and 290,000–650,000 deaths worldwide [11]. Antivirals are available, most prominently inhibitors of the neuraminidase (NA) or the ion channel matrix protein 2 (M2). However, they have only a modest impact on disease, and their use can select for resistant strains [1, 6].

FLUAV particles have a lipid bilayer containing the envelope proteins HA (hemagglutinin) and NA and the proton channel protein M2 [3, 8]. Inside the particles are the matrix protein M1 and eight nucleocapsids consisting of the negative-stranded RNA genome, the nucleoprotein (NP), and the associated RNA polymerase complex (subunits PB1, PB2, and PA). The eight genomic segments encode the structural proteins and the auxiliary proteins NS1 and NEP. Viral transcription and replication occur in the nucleus and are regulated by the partially complementary 5’ and 3’ untranslated regions, which serve as a promoter for the PB1/PB2/PA polymerase [3].

Recently, Patel and Kukol predicted by *in silico* analysis that a Food and Drug Administration (FDA)-approved drug against schizophrenia, paliperidone [9], may be a novel antiviral against FLUAV [7]. The paliperidone molecule was predicted by modeling to interact with an evolutionarily conserved region of the viral polymerase subunit PB2 that also contains NP-binding residues [7].

We evaluated the effect of paliperidone on multistep replication of three FLUAV H1N1 strains: A/PuertoRico/8/1934 (A/PR/8/34), A/Hamburg/4/2009 (A/HAM/2009), and A/WSN/1933 (A/WSN/33). These virus strains were originally isolated from human cases in the year indicated in their name and cultivated in cell lines since then (http://www.fludb.org; [13]). These isolates were chosen because they are widely used in influenza research, and A/HAM/2009, which was the cause of the most recent influenza pandemic, is still circulating. As a cell system, we initially used the highly FLUAV-permissive MDCK II cells (ATCC MDCK.2 CRL-2936; [3]) cultivated in Dulbecco's modified Eagle's medium (DMEM) with 10% fetal bovine serum (FBS) as described previously [10].

Cells were infected with virus suspended in infection medium (DMEM containing 0.2% BSA and 1 μg of L-1-tosylamido-2-phenylethyl chloromethyl ketone (TPCK)-treated trypsin) per ml at a multiplicity of infection (MOI) of 0.01 as described [10] and treated 1 h later with medium containing 10 µg of paliperidone (Sigma Aldrich; P0099; stocks dissolved in DMSO) per ml. Supernatants were harvested at 8, 24, 48, and 72 hours postinfection (p.i.). This concentration of paliperidone was chosen firstly because it was below the toxicity threshold (Supplementary Fig. S1), and secondly because it was also used by Yang *et al.* for their pharmacological studies in human brain cancer cells [12].

Virus titration of the supernatants by plaque assay [5] showed that paliperidone indeed had an effect against strain A/PR/8/34, which was, however, modest and restricted to the 8 h p.i. time point (Fig. 1A). Moreover, neither of the other viruses was inhibited (Fig. 1B and C). Similar results were obtained using the human alveolar basal epithelial cell line A549, which is also often used in FLUAV research (Supplementary Fig. S2).

**Fig. 1:**
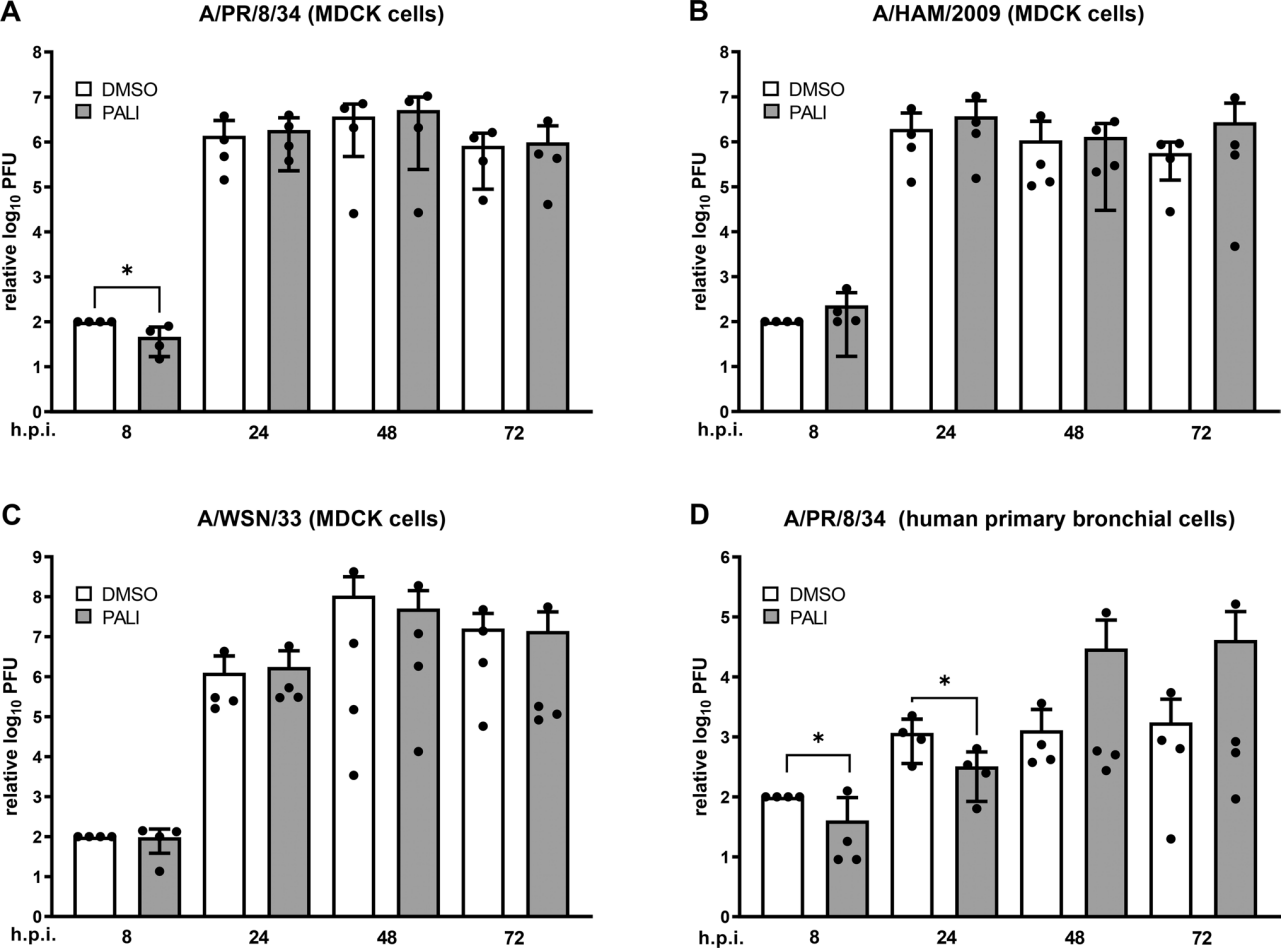
Paliperidone and the FLUAV replication cycle. MDCK II cells (A to C) or human primary bronchial cells (D) were infected with the indicated viruses at an MOI of 0.01 or 1 and treated 1 h later with medium containing 10 µg of paliperidone per ml or the solvent DMSO as a control. At the time points indicated, supernatant samples were collected and virus titers were determined. Control values at the earliest measurable time point were set to 100%. Mean values and standard deviations from four independent experiments are shown. Statistical testing was done using a one-tailed unpaired Student’s *t*-test. * indicates *p* < 0.05; all other comparisons had *p*-values > 0.05.

Thus, A/PR/8/34 was the only strain with some transient sensitivity to paliperidone. To investigate this further, we employed primary human bronchial cells as a system to more closely represent the *in vivo* situation [2]. These cells were prepared from cryopreserved normal human bronchial epithelial cells of non-smoking donors (Lonza; CC-2540). They were seeded in the undifferentiated state and grown in a mixture of DMEM and Bronchial Epithelial Cell Growth Medium (Lonza; CC-3170) supplemented with 75 nM retinoic acid with medium exchange every second day. After reaching confluence, the cells were cultivated under air-liquid conditions for at least four additional weeks for full differentiation into pseudostratified human airway epithelia. Medium from the basolateral compartment was renewed every second day, and the apical surface was washed weekly with PBS (Thermo Fisher). Before infection, the cells were washed at least twice (to remove mucus) with phosphate-buffered saline (PBS) and inoculated for 1 h with virus in infection medium. After further incubation at 37°C, supernatants were harvested at the indicated time points. As shown in Figure 1D, paliperidone was also able to retard A/PR/8/34 replication in primary human bronchial epithelial cells. The antiviral effect was detectable at 8 h and at 24 h p.i., but not later. In fact, at the 48 h and 72 h time points, there appeared to be a slight trend towards higher virus titers under paliperidone treatment, but this was caused by single outliers and was not statistically significant.

Patel and Kukol predicted that paliperidone docks into a pocket on PB2 that is evolutionary conserved and overlaps with a domain that is important for NP binding [7]. Therefore, we examined by co-immunoprecipitation assays whether the compound could affect the formation of the PB2-NP complex. A549 cells were infected with strain A/PR/8/34 for 6 h in the presence or absence of paliperidone at an MOI of 1 and scraped off the dishes in PBS, followed by centrifugation for 5 min at 800 *g*. Pellets were resuspended in RIPA buffer (50 mM Tris-HCl pH 7.5, 0.15 M NaCl, 1% NP40) containing protease inhibitors (cOmplete Protease Inhibitor Cocktail, Roche, 04693116001) and incubated for 10 min at 4°C. After further centrifugation (10,000 *g*, 10 min, 4°C) supernatants were immunoprecipitated using a mouse monoclonal anti-p53 antibody (Calbiochem, OP43) and rabbit anti-NP (Genetex GTX125989) and anti-PB2 (Genetex GTX125926) polyclonal antibodies as described [10]. Subsequently, the immunoprecipitates were probed with mouse monoclonal anti-p53 antibody (1:1000), rabbit polyclonal anti-NP (1:1000) and anti-PB2 (1:200) antibodies, and anti-beta tubulin antibody (Abcam, ab6046, (1:1000) using polyvinylidene difluoride membranes and a semidry blot transfer apparatus (Bio-Rad). As shown in Figure 2A, both viral proteins were detectable in the input control, as expected, and paliperidone treatment had reduced their signals. Moreover, NP immunoprecipitations contained PB2, and vice versa, whereas both negative controls (1. uninfected cells, 2. immunoprecipitation with an anti-p53 antibody) remained blank. Interestingly, application of paliperidone resulted in less PB2 in the NP immunoprecipitates, and conversely, there was less NP in the PB2 immunoprecipitates. Along these lines, quantification of the immunoblot signals from independent experimental replicates, normalized to the signal of the respective immunoprecipitated viral protein, revealed that paliperidone reduces the binding between PB2 and NP, whereas the ratio of the two proteins in the input lysates remained unchanged (Fig. 2B). The PB2:NP ratios in the input lysates were unaffected by paliperidone because the levels of both proteins were reduced in a similar manner. By contrast, the PB2:NP and the NP:PB2 ratios were diminished by paliperidone due to the impact on the mutual binding (see Fig. 2B). Interestingly, when paliperidone was added later in infection, it was no longer able to disrupt PB2-NP binding (Supplementary Fig. S3A and B). Taken together, we could verify the *in silico* prediction [7] that paliperidone disturbs the interaction of FLUAV PB2 with NP. Paliperidone thereby affects the initial formation of the PB2-NP complex but cannot promote disassembly of complex once it is formed.

**Fig. 2:**
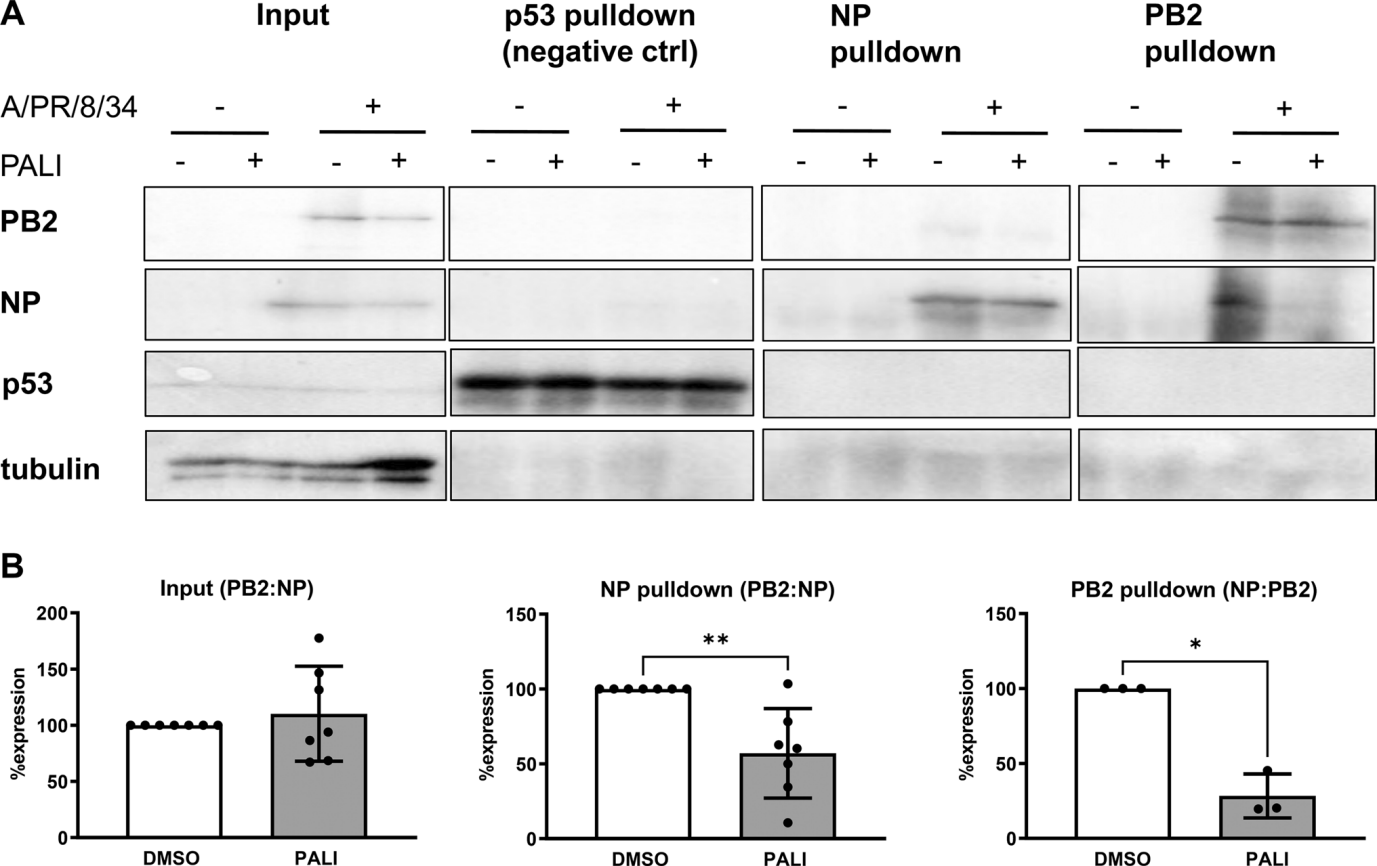
Influence of paliperidone on PB2-NP binding. Human A549 cells were infected at an MOI of 1 and treated 1 h later with 10 µg of paliperidone per ml or the solvent DMSO as a control. A further 6 h later, total protein was extracted, and 10% of the lysate was used as input, while p53 pulldown was used as a negative control. (A) Representative immunoblots (B) Ratios of quantified immunoblot signals for PB2 and NP. DMSO control values for infected input cells were set as 100%. Mean values and standard deviations from seven (input and NP pulldown) or three (PB2 pulldown) independent experiments are shown. *: *p* < 0.05. Please note that immunoprecipitations were initially only performed with an anti-NP antibody, but for the last three experiments, they were extended with an anti-PB2 antibody in parallel. Statistical testing was performed as indicated for Figure 1.

We wondered whether the interference with polymerase assembly may have repercussions on viral RNA synthesis. To address this question, we measured levels of viral positive-strand RNA derived from genome segment 7 in cells that were infected for 6 h with strain A/PR/8/34. Total RNA was isolated from cells using an RNeasy Mini Kit (QIAGEN), and 10 ng of this preparation was analyzed by RT-qPCR in a StepOne Real-Time PCR System (Applied Biosystems). Reverse transcription was performed using PrimeScript RT Master Mix (Takara RR036A), and polymerase chain reaction was performed using SYBR Premix Ex Taq (Tli RNase H Plus, Takara RR420A). Cellular GAPDH RNA was quantified using QuantiTect primer QT01192646, and FLUAV segment 7 sequences encoding the M protein were detected using the (−) strand-specific forward primer 5’-GGA CTG CAG CGT AGA CGC TT -3’ and the (+) strand-specific reverse primer 5’- CAT CCT GTT GTA TAT GAG GCC CAT -3’ (also used for reverse transcription of viral RNA). Values were normalized against GAPDH using the ΔΔCT method [4]. The RT-qPCR analysis, shown in Figure 3A, demonstrate that paliperidone indeed suppressed positive-strand RNA synthesis of A/PR8/34 by approximately 50%, although the variation was quite high. Also, immunoblot analysis of cell-borne proteins showed a reduction in the levels of NP, one of the most abundant structural proteins of FLUAV (Fig. 3B). Thus, paliperidone shows some effectiveness against early RNA and protein synthesis by FLUAV A/PR/8/34.

**Fig. 3:**
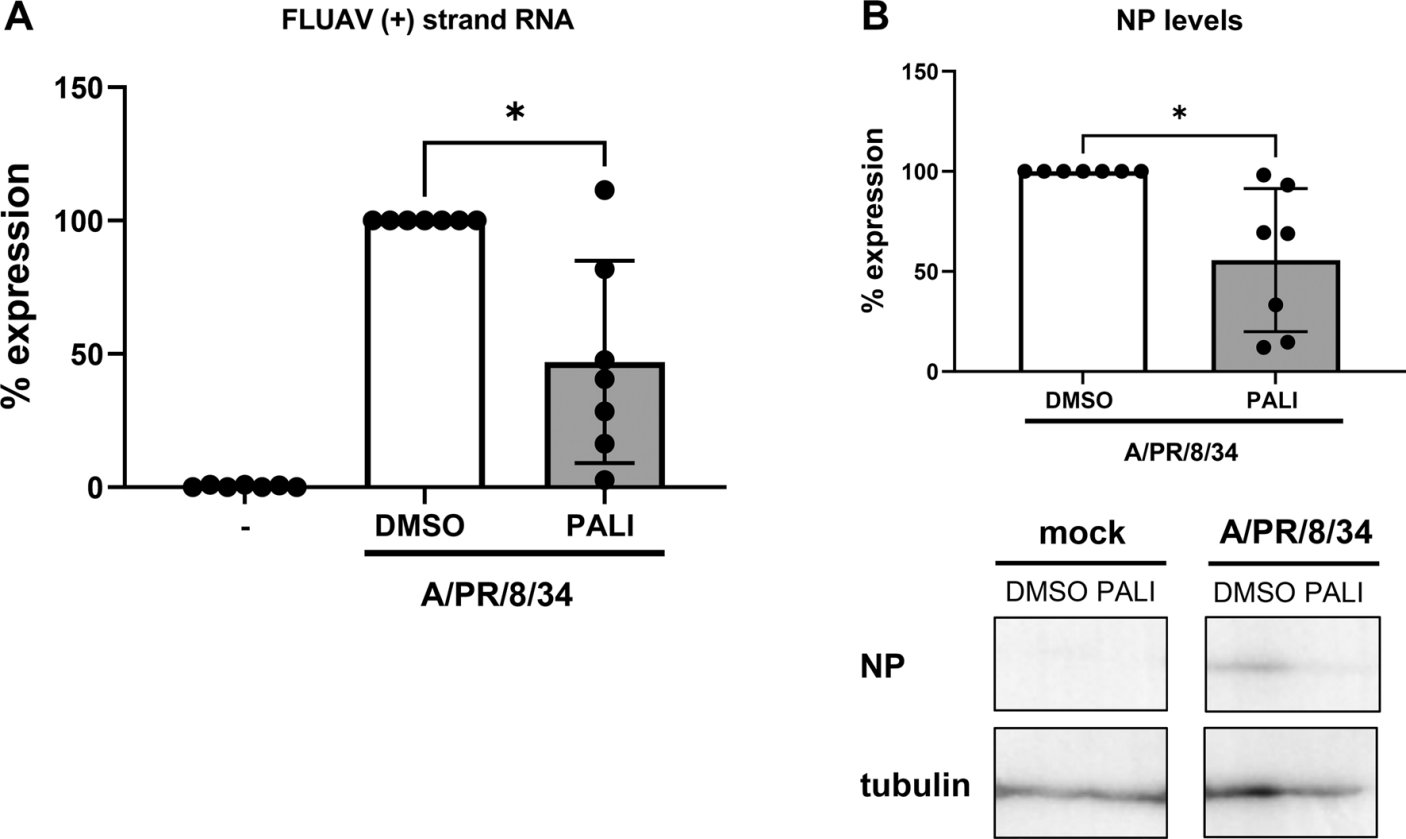
Effect of paliperidone on the early stage of A/PR/8/34 replication. (A) Human A549 cells were infected at an MOI of 1 and treated 1 h later with 10 µg of paliperidone per ml or the solvent DMSO as a control. A further 6 h later, total RNA was isolated, and levels of positive-strand viral RNA derived from genome segment 7 were determined by real-time RT-PCR. (B) A549 cells were infected and treated as described for panel A, but proteins were extracted and analyzed by immunoblotting to detect viral NP and cellular β-tubulin. Immunoblot signals from seven independent experiments were quantified, the FLUAV NP levels were normalized to β-tubulin, and the DMSO control values of infected cells were set as 100% (top panel). A representative immunoblot is shown in the bottom panel. In panels A and B, mean values and standard deviations from seven independent experiments are shown. Statistical testing was performed as indicated for Figure 1. *, *p* < 0.05

Based on their *in silico* analysis, Patel and Kukol suggested using the FDA-approved schizophrenia drug paliperidone [9] as a new lead compound against FLUAV [7]. We addressed this suggestion using different H1N1 strains of FLUAV. Indeed, for A/PR/8/34, paliperidone was found to affect RNA and protein synthesis as well as progeny particle production early in the infection cycle, also in a relevant *ex vivo* system based on human primary bronchial cells. However, other H1N1 strains were not affected at all, and also for A/PR/8/34, the antiviral effect waned upon longer infection periods. Mechanistically, we demonstrated that paliperidone indeed disturbs the interaction between the A/PR/8/34 polymerase subunit PB2 and the nucleoprotein NP, which is consistent with the prediction that the compound docks onto residues on PB2 that are involved in NP binding [7]. Moreover, although the predicted paliperidone docking site on PB2 (G222, S225 to Q257, E525, I529) is highly conserved among the three strains, there are differences at positions 225 and 250 (Supplementary Fig. S4) that could potentially influence the binding of paliperidone, NP, or both. Moreover, on the NP there are also slight sequence differences in the PB2-binding site (Supplementary Fig. S5) that might affect NP-PB2 binding, and hence the susceptibility to paliperidone. In addition, there might be other regions on PB2 and/or NP that are involved but were not detected by the *in silico* docking. In any case, however, our data confirm the *in silico* predictions but suggest the need for caution in using paliperidone as a starting point for the development of new anti-influenza drugs, as extensive optimizations will certainly be required.

## Supplementary Information

Below is the link to the electronic supplementary material.

Supplementary file1 (PDF 632 kb)

## Data Availability

All data are included in the manuscript and the supplemental information file.
