# Supplementary material for: Influenza A virus strain PR/8/34, but neither HAM/2009 nor WSN/33, is transiently inhibited by the PB2-targeting drug paliperidone
Source: Arch Virol. 2023 Jan 13;168(2):63. doi: 10.1007/s00705-022-05696-0 (PMC9839214; doi:10.1007/s00705-022-05696-0)
Supplement: Supplementary file 1 — Supplementary file1 (PDF 632 kb) [file 705_2022_5696_MOESM1_ESM.pdf]

Supplementary information

Influenza A virus strain PR/8/34, but neither HAM/2009 nor WSN/33, is transiently inhibited by the PB2-targeting drug Paliperidone

Georgios-Dimitrios Panagiotidis [1], Christin Müller [2,3], Marco Binder [4] and Friedemann Weber [1,3]*

1 Institute for Virology, FB10-Veterinary Medicine, Justus-Liebig University, D-35392 Giessen, Germany

2 Institute for Medical Virology, FB11- Medicine, Justus-Liebig University, D-35392 Giessen, Germany

3 German Center for Infection Research (DZIF), partner site Giessen, Germany

4 Research Group "Dynamics of early viral infection and the innate antiviral response", Division "Virus-Associated Carcinogenesis", German Cancer Research Center (DKFZ), Heidelberg, Germany

* Correspondence: friedemann.weber@vetmed.uni-giessen.de; Tel.: +49-641 9938350

Figure S1

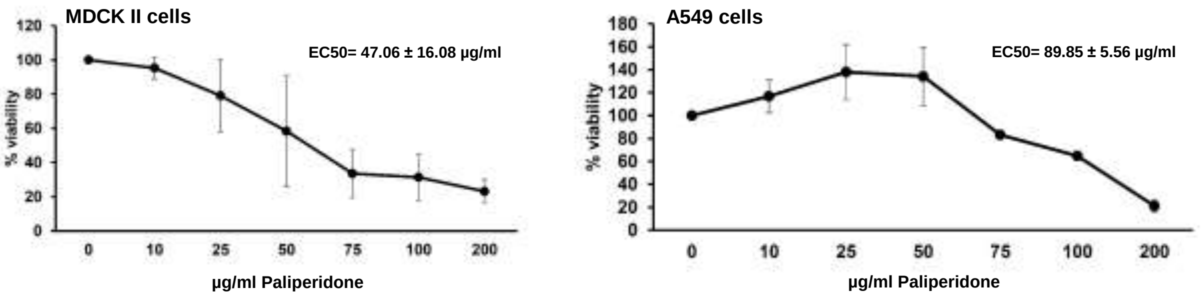

Effect of Paliperidone on cell viability. Cells were treated with the indicated amounts of Paliperidone 48 h (MDCK II) or for 24 h (A459) and then subjected to the neutral red uptake assay as described by Repetto et al. (Nat Protoc. 2008;3(7):1125-31. doi: 10.1038/nprot.2008.75.) to estimate cell viability. Mean values and standard deviations from 3 independent experiments are shown. EC50s (effective concentrations for 50% viability) are indicated.

Figure S2

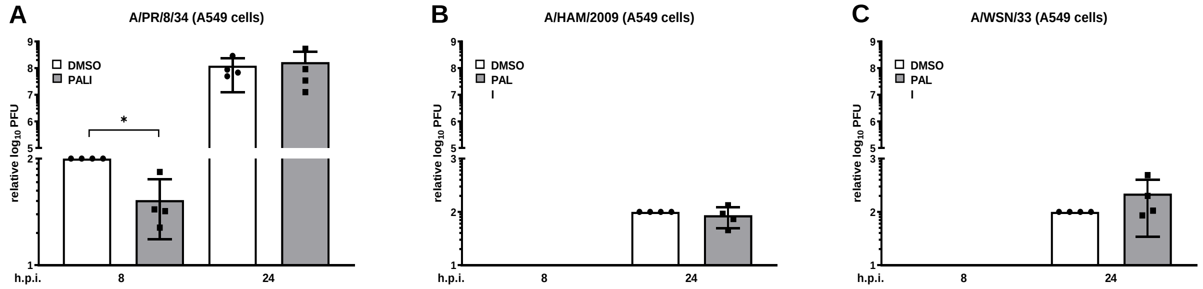

Paliperidone and the FLUAV replication cycle in human A549 cells. Human A549 cells (ATCC A549 CRM-CCL-185), cultivated in DMEM with 10% FBS were infected at an MOI of 0.01 and 1 h later treated with 10 µg/ml Paliperidone or the solvent DMSO as control. At the time points indicated, supernatants were isolated and virus titers determined. Values of the earliest measurable control time point were set to 100%. Mean values and standard deviations from 4 independent experiments are shown. Statistical testing was done using one-tailed unpaired Student's t test. * indicates p<0,05, all other comparisons had p values > 0.05. At later time points the cells underwent cytopathic effect, thus not permitting reliable titration of supernatants.

Figure S3

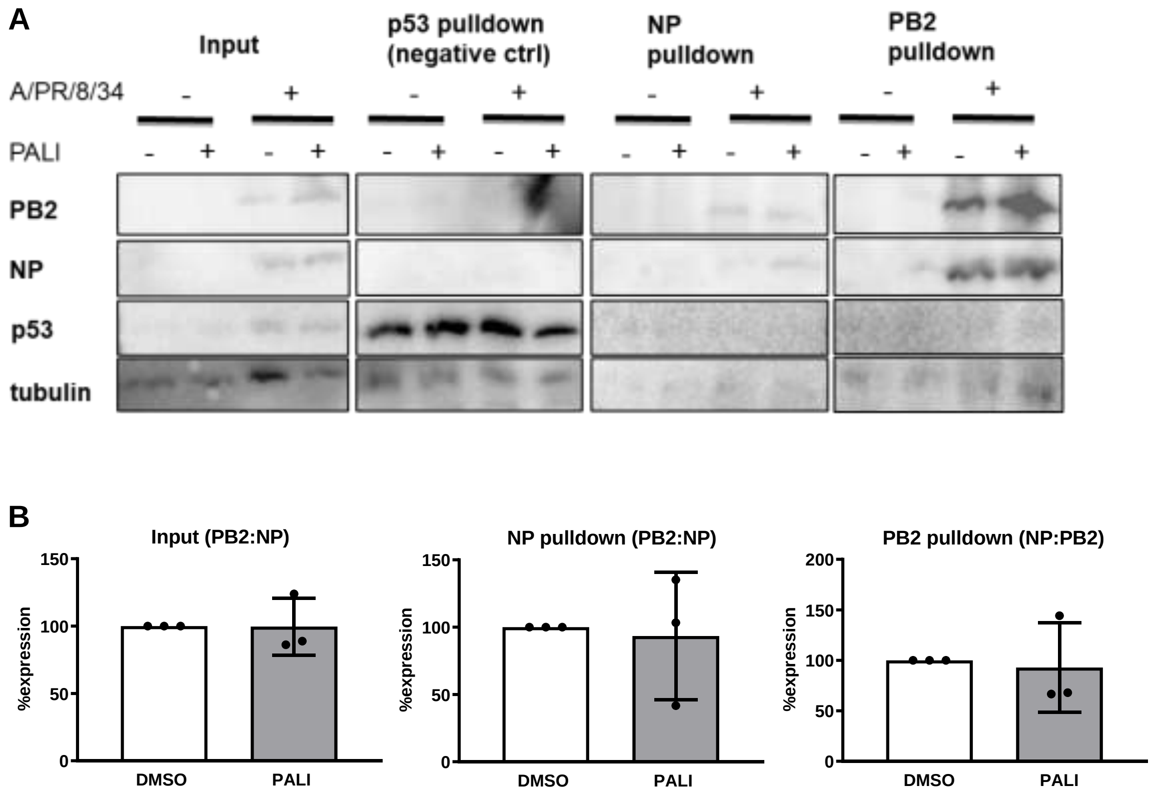

Influence of Paliperidone on a preassembled PB2-NP complex. Human A549 cells were infected, incubated, lysed and assayed by immunoprecipitations as described for figure 2. Ten µg/ml Paliperidone (or the solvent DMSO as control), however, were added to the lysates, which were then incubated for 60 min before immunoprecipitations took place. (A) Immunoblots and (B) ratios of quantified immunoblot signals. All statistical analyses had p values > 0.05.

Figure S4

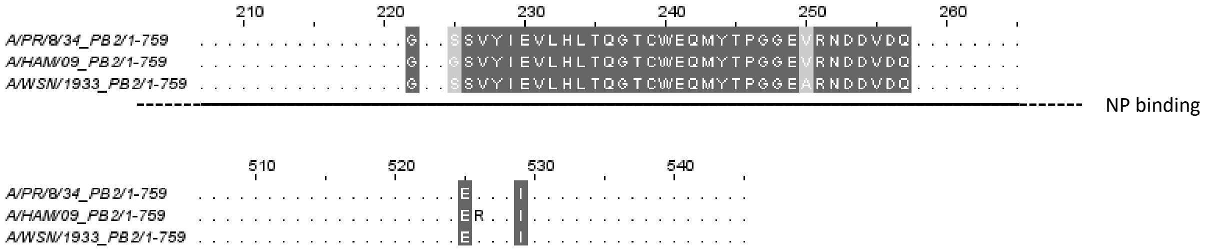

Evolutionary conservation of the proposed Paliperidone-binding site of PB2. Alignment of PB2 amino acid sequences that were in silico predicted to be critical for Paliperidone binding (Patel and Kukol, Virology. 2017 Sep;509:112-120. doi: 10.1016/j.virol.2017.06.009). PB2 sequences for FLUAV strains A/PR/8/34 H1N1 (Sequence ID AGQ48061), A/HAM/2009 H1N1 (ACR10217.1), and A/WSN/33 H1N1 (AAA43611.1) are shown. The putative Paliperidone-interacting PB2 residues are highlighted depending on their degree of evolutionary conservation (Dark grey: highly conserved, light grey: less conserved). Dots in the alignment indicate highly conserved residues outside of the predicted Paliperidone binding site. The black line underneath the upper alignment illustrates that these sequences are involved in NP binding (Poole et al., Virology. 2004 Mar 30;321(1):120-33. doi: 10.1016/j.virol.2003.12.022): Sequence information was obtained from NCBI Protein and analyzed with Clustal Omega (Sievers and Higgins, Protein Sci. 2018 Jan;27(1):135-145. doi: 10.1002/pro.3290).

Figure S5

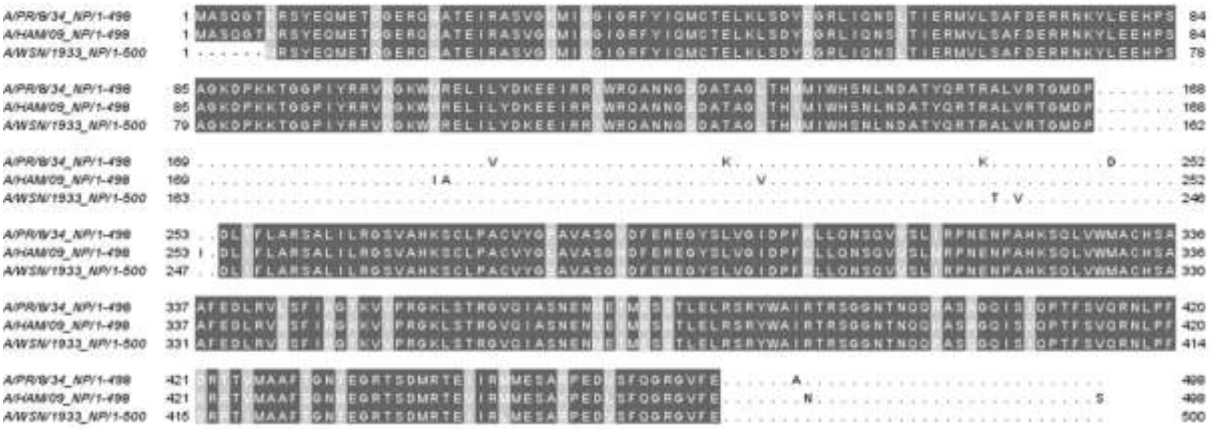

Evolutionary conservation of the PB2-binding sites on NP. Alignment of NP amino acid sequences according to Biswas et al (J Virol. 1998 Jul;72(7):5493-501. doi: 10.1128/JVI.72.7.5493-5501.1998). Legends and methods are as for Fig. S4. Sequence IDs are NP_040982.1 (A/PR/8/34), ADL41174.1 (A/HAM/2009), and 4DYT_C (A/WSN/33).
